# The Surgical Treatment of Duodenal Ulcer

**Published:** 1907-08-24

**Authors:** Leonard A. Bidwell

**Affiliations:** Dean of the Post-Graduate College, and Surgeon to the West London Hospital


### Hospital Clinics.

### THE SURGICAL TREATMENT OF DUODENAL ULCER.

By LEONARD A. BIDWELL, F.R.C.S.Eng., Dean of the Post-Graduate College, and Surgeon to the West London Hospital.

### Symptoms and Diagnosis.

The first point to consider in duodenal ulcer is the diagnosis, but although considerable attention has been paid to this subject during the past few years, it is still far from simple. Duodenal ulcer is more common in man than is gastric ulcer; and it is more common in man than in woman. Duodenal ulcer is more common in middle age than in youth, whereas gastric ulcer is common in the young.

Vomiting is very rarely present in the early stages of duodenal ulcer; it occurs in the late stages, when cicatricial contraction exists. In gastric ulcer vomiting is one of the first symptoms. The symptom which induces a patient with a duodenal ulcer to seek advice is pain, and pain is often the only symptom which will be apparent until a late stage of the case; it is of a dull aching character, and occurs some time after food, usually from one to four hours. The pain steadily gets worse; it comes on a shorter time after food and lasts longer. At first it is relieved by taking food. Another curious symptom of duodenal ulcer is "hunger pain." It is a sort of irresistible desire for food, a violent feeling of hunger. Immediately food is taken the pain is diminished, to come on again shortly. This is very seldom found in gastric ulcer. Nausea and water brash are common, though vomiting is rare. In about half of the cases which I have seen, there has been a history of hæmorrhage, either in the form of hæmatemesis or of melæna. When hæmatemesis occurs the amount of bleeding is not very profuse, but in melæna the bleeding is sometimes very copious indeed, and so severe that the patient suddenly becomes faint and pale; while on the following day a copious motion of digested blood is passed per rectum. We have to remember also that a great number, nearly a quarter, of cases of duodenal ulcer are associated with gastric ulcer; difficulties in diagnosis arise from this cause.

### Illustrative Cases.

The best way to illustrate the symptoms of duodenal ulcer will be to describe some of the cases which I have seen in consultation with physicians. The first case was that of a man, fifty-five years of age, who had suffered from flatulent dyspepsia for seventeen years. About two years before I saw him he began to get anæmic and to lose strength. He was treated for pernicious anæmia. Some blood was found in the stools, but that did not make his physicians suspect duodenal ulcer. He remained much the same for eighteen months, when he had a very severe hæmatemesis, and the question of gastric ulcer was discussed. There was no hunger pain nor pain after food. Ten days before my visit, he suddenly became pulseless and collapsed and passed a copious black motion next day. He was put on rectal feeding, given nothing by the mouth, but eight days later a similar hæmorrhage occurred after a dose of castor oil. When I saw him he was sallow and emaciated with a retracted abdomen and a very soft pulse, and he complained of some tenderness on pressure in the middle line, just above the umbilicus. In this case we could not assist diagnosis by passing a stomach-tube, on account of the risk of setting up fresh bleeding. The condition was exceedingly serious, and I advised a delay of four days before opening the abdomen. The operation was performed four days after the second hæmorrhage, a considerable amount of induration was found in the second part of the duodenum, and an ulcer which extended three-quarters round the gut; a posterior gastro-enterostomy was performed. This case did not have many of the usual symptoms of duodenal ulcer; practically the only symptom was hæmorrhage.

The second case was a gentleman, aged forty-five, who had suffered from attacks of hyperchlorhydria for about twenty years. These attacks of excessive acidity of the gastric juice caused a certain amount of pain and were followed by water brash. He had an intense craving for food and curious nerve pains, with a terrible feeling of misfortune hanging over him. He became so neurasthenic that he had to give up his business in the city. Six months before I saw him the attacks of vomiting became more severe, and the vomit contained a large amount of free hydrochloric acid. Lavage had been tried with benefit, but the Weir-Mitchell treatment produced no good effect. This patient had neither melæna nor hæmatemesis. The stomach was slightly dilated, but did not extend below the umbilicus. A stomach tube was passed and some contents withdrawn; the stomach was then blown up with air through a Higginson's syringe, and I was able to define the lower border of the stomach. This method of distending the stomach is preferable to the method of giving a seidlitz powder in two parts as in the latter method a large amount of carbonic acid gas is generated, which produces spasm of the pylorus, and prevents the gas from escaping onwards and so causes pain. With a stomach tube the stomach can be distended as much as is required, and air does not produce any spasm. The only objection to the method is that a stomach tube has to be passed. To return to the history of this patient. He had intervals of complete freedom from pain, and he was then able to enjoy himself and felt perfectly well; but these intervals gradually became shorter. On these symptoms the physicians recommended exploration as a last resource. The reason why they suspected duodenal ulcer was that he had very marked hunger pain and a very considerable degree of hydrochlorhydria. I opened the abdomen and found an indurated ulcer in the second part of the duodenum, and performed posterior gastro-enterostomy.

### Two Other Cases.

The next case is one in which I think the symptoms are very characteristic. A lady, aged forty-three years, had suffered from pain after food for seven or eight years; vomiting was very occasional, and there had never been any hæmatemesis. The pain came on usually two hours after food—sometimes earlier—and was relieved by taking food. She suffered from flatulence and became emaciated. She was never free from pain for more than two days. The stomach was blown up and found to be dilated and extended below the umbilicus. At the operation a large indurated ulcer was found on the posterior surface of the first part of the duodenum, and, being close to the pylorus, it had produced a cicatricial stricture, and thus had caused dilatation of the stomach.

The last case was a man, aged fifty-five, who gave a history of pain after food for seven months, but no sickness or nausea until a week before I saw him, when he started "coffee ground" vomiting. He vomited everything he took, so that he was afraid to take even liquid food. The stomach was dilated and extended one inch below the umbilicus. The age of the patient and the history of seven months' pain after food, followed by vomiting of "coffee-ground" material, made one fear that it was a case of malignant disease. An examination of the stomach-contents showed that free hydrochloric acid was present, so the prognosis was hopeful. At the operation an ulcer was found in the usual place in the duodenum, and complete relief was afforded by a gastro-enterostomy.

These cases will serve to illustrate the symptoms. In all of them we found the condition of hyperchlorhydria. This is demonstrated if the contents of the stomach are tested with a solution of phloroglucin and vanillin. Put a drop of the solution and a drop of the stomach contents on a white plate and allow the two drops to coalesce, and heat over a spirit lamp; if hydrochloric acid is present a pink colour is produced, the depth depending upon the amount of acid present. If none is present a brown colour is obtained. This test will often prevent the mistake of diagnosing a simple duodenal ulcer as malignant disease. Fortunately, malignant disease is rare in the duodenum, although common in the stomach as the result of gastric ulcer. If the ulcer is in the duodenum it is most probably not malignant.

### Indications for Operations.

In former days the advanced surgeon used to recommend operations in duodenal ulcer only after rupture, repeated hæmorrhages, or cicatricial contraction. I have quoted to you examples of operation for repeated hæmorrhages and for cicatricial contraction. In the case of rupture there can be no question as to the necessity for operation. In cases of rupture of a duodenal ulcer the previous history may be very indefinite, because rupture often takes place early in the history of the ulcer, before it has produced hæmorrhage or cicatricial contraction—before, in fact, it has produced any symptoms except pain and flatulence. The symptoms of rupture of a duodenal ulcer are very acute, since most cases lead to general peritonitis and extravasation of duodenal contents into the abdomen. About three months ago a woman thirty years of age was admitted into hospital, complaining of acute pain in the abdomen, which had come on suddenly after indefinite indigestion of some standing. This acute pain came on about five o'clock in the morning. She saw a doctor, who did not think that it would be serious, and gave her a little opium, which made her more comfortable. In the evening she had a great deal more pain, the abdomen became rigid, and she was sent to the hospital. The abdomen was slightly distended, with marked rigidity of the upper half of the right rectus muscle—a very important sign of rupture of a duodenal ulcer. Practically there are only two conditions which will produce that marked rigidity of the right rectus—namely, rupture of a duodenal ulcer and an inflamed gall-bladder or duct. In this case the acuteness of the onset and the existence of some dullness in the flanks were important as pointing to duodenal ulcer rather than gall-stone trouble. She had also a rapid pulse of about 130, and a temperature raised slightly above normal. When the abdomen was opened an ulcer was found in the second part of the duodenum. It had ruptured, and the duodenal contents had escaped freely into the general peritoneal cavity.

### Sutures and Drainage.

In order to close the rupture we put in a double row of Halsted's stitches a quarter of an inch from the edge of the ulcer. In bringing the edges of a ruptured ulcer together it is important to ensure that the scar is in the transverse and not in the vertical diameter of the bowel. It is essential to thoroughly clean away the extravasated duodenal contents, specially between the liver and the diaphragm and between the spleen and the diaphragm. These spaces must be thoroughly swabbed out with sterilised mops, and a counter-opening must be made posteriorly, below the ribs, on each side.

A stab puncture is made through the skin close to the erector spinæ muscle, and then a pair of strong pedicle forceps are pushed through from the outside; they enter the abdomen internal to the ascending colon, of course avoiding the kidney. After pushing the forceps through the stab puncture in the loin, the point is made to present in the abdominal wound, and a drainage tube is caught by the blades and drawn out through the loin, leaving its end projecting about half an inch from the parietal peritoneum. If there has been much extravasation, the pelvis must also be drained by making an incision just above the pubes and passing the tube down from the abdomen to the pelvis. After the drainage tubes have been inserted the abdomen may be washed out with saline solution. Never on any account use anything but this. Washing out the abdomen even with plain water or before the drainage tubes have been put in may do harm. Plain water injures the sensitive cells of the peritoneum, and if you wash out before the drainage tubes are in you are liable to wash the irritating material into the remoter recesses of the peritoneum. You must be certain that you have a free escape for any fluid before you attempt to wash. If a ruptured ulcer is treated on these lines, by closing the ulcer and providing free drainage, and provided that it is done within twelve hours of the rupture, the result should be satisfactory. It is now the rule rather than the exception for the cases to recover, mostly due, I believe, to the method of drainage and to the avoidance of antiseptics or plain water. Antiseptics were commonly employed in former days, and their use was almost certainly fatal.

With the old indications for operations all will agree—namely, rupture, hæmorrhage, and cicatricial contraction—but we go much farther nowadays, and recommend operation in all cases of duodenal ulcer in which the diagnosis can be made out. Medical treatment is practically useless in the treatment of duodenal ulcer, since whether food is taken or not the ulcer must always be irritated by the acid gastric juice passing over it.

### The Site of Ulceration.

It is interesting to notice that the position of the ulcer is nearly always just above the inlet of the bile duct, and so it occurs in a portion of intestinal mucous membrane which is bathed in gastric juice before that fluid meets with and is neutralised by bile. I have never come across a duodenal ulcer which has started below the bile duct; it is either opposite to or above it. The probable explanation of the relief of pain in the case of duodenal ulcer after taking food is that immediately after food is taken the pylorus contracts, and remains contracted till the first stage of digestion is over, and thus prevents the gastric juice from leaving the stomach and irritating the ulcer. When the pylorus opens at the end of that stage of digestion the gastric juice and food pass over the ulcer, and then pain commences. The "hunger pain" seems to be caused simply by an irritation of the ulcer by gastric juice.

### Hæmorrhage.

Considering the position of the ulcer, it is natural that hæmorrhage should take a downward course and appear as melæna. Those cases in which it occurs as hæmatemesis are cases in which the bleeding occurs at the time when the stomach is empty and the pylorus dilated. As soon as the food comes into the stomach the pylorus becomes closed, so that if the bleeding occurs after food melæna is more likely than hæmatemesis. If it occurs during fasting, hæmatemesis will be probable, and not melæna. A duodenal ulcer which does not perforate may heal up, and in so doing may cause cicatricial contraction. When this occurs the stomach becomes dilated, and persistent vomiting is an important sign. Sometimes one sees hypertrophy of the stomach very much like that of the bladder in a case of stricture of the urethra. I have operated upon cases of duodenal ulcer in which the stomach walls have been greatly thickened, but in which there was very little dilatation of the stomach.

When dilatation is fully established an important sign is the vomiting of an apparently larger quantity of food than has been taken. Some of the vomited matter will probably be undigested portions of food which have been taken two or three days, or even months, before. The most extreme case was one quoted by Moynihan of a person who had eaten plums in the August of one year and in whose stomach plum stones were found in May of the following year. On washing out the stomach, therefore, you may find evidence of this stasis of food, and it is quite common in dilatation to find foodstuff which has remained in the stomach for a fortnight.

### The Operation.

The operation usually performed is that of gastro-enterostomy, except in cases of a perforated ulcer, when closure of the perforation has to be performed also. By gastro-enterostomy the gastric contents are prevented from passing over the ulcer, and in nearly every case it is a satisfactory method of treatment. The point aimed at is drainage of the most dependent portion of the stomach. In a dilated stomach the plyorus is displaced, and is no longer the lowest part of the stomach. The pyloric antrum when distended is on a lower level than the pylorus. In cases where there is no pyloric obstruction, it is necessary to prevent the gastric contents from reaching the pylorus, and to do this it is essential to make the opening at the lowest part of the stomach. The opening must be made in a certain direction —namely, downwards and to the right—so that the waves of the contraction in the piece of jejunum applied to the stomach shall be in the same direction as the waves of peristaltic contraction in the stomach. The opening should be made in the direction of these movements, so that the food, on entering the jejunum, will be passed on towards the ileum. The opening in the jejunum should be made as close as possible to its commencement, close to the ligament of Treitz. This is more or less a new idea.

In the old days it was considered better to leave a loop of jejunum before attaching it to the stomach, with the idea of relieving the strain upon the stitches. This method was apt to be followed by vomiting, due to the formation of a vicious circle. In my earlier cases I have had experience of this condition, but not recently. The vicious circle was cured immediately by entero-enterostomy.

### Results of Operation.

I have now operated on a considerable number of cases of duodenal ulcer, and I have not lost any cases except after perforation, and the immediate results have been excellent. The great majority of cases are permanently cured by the operation.

One warning which I give patients now is that for a few months after a gastro-enterostomy they may not be free from discomfort and indigestion. It takes that time for the stomach to settle down to its new conditions. The recurrence of pain is transitory and passes off. The condition of the patient a year after the operation is the important thing, and that, as a rule, is absolutely satisfactory.

